# Navigating the Maze of Social Media Disinformation on Psychiatric Illness and Charting Paths to Reliable Information for Mental Health Professionals: Observational Study of TikTok Videos

**DOI:** 10.2196/64225

**Published:** 2025-06-18

**Authors:** Alexandre Hudon, Keith Perry, Anne-Sophie Plate, Alexis Doucet, Laurence Ducharme, Orielle Djona, Constanza Testart Aguirre, Gabrielle Evoy

**Affiliations:** 1 Department of Psychiatry and Addictology, Université de Montréal, Montréal, QC, Canada; 2 Department of Psychiatry, Institut national de psychiatrie légale Philippe-Pinel, Montréal, QC, Canada; 3 Department of Psychiatry, Institut universitaire en santé mentale de Montréal, 7401 Rue Hochelaga, Montréal, QC, H1N 3M5, Canada, 1 5149954842; 4 Centre de recherche de l'Institut universitaire en santé mentale de Montréal, Montréal, QC, Canada

**Keywords:** psychiatry, mental health, social media, mental illness, psychoeducation, disinformation, misinformation, psychology, psychotherapy, artificial intelligence, phenomenon, professional, observational analysis, stigma, educational video, TikTok, algorithm

## Abstract

**Background:**

Disinformation on social media can seriously affect mental health by spreading false information, increasing anxiety, stress, and confusion in vulnerable individuals, as well as perpetuating stigma. This flood of misleading content can undermine trust in reliable sources and heighten feelings of isolation and helplessness among users.

**Objective:**

This study aimed to explore the phenomenon of disinformation about mental health on social media and provide recommendations to mental health professionals that would use social media platforms to create educational videos about mental health topics.

**Methods:**

A comprehensive analysis conducted on 1000 TikTok videos from more than 16 countries, available in English, French, and Spanish, covering 26 mental health topics. The data collection was conducted using a framework on disinformation and social media. A multilayered perceptron algorithm was used to identify factors predicting disinformation. Recommendations to health professionals about the creation of informative mental health videos were designed as per the data collected.

**Results:**

Disinformation was predominantly found in videos about neurodevelopment, mental health, personality disorders, suicide, psychotic disorders, and treatment. A machine learning model identified weak predictors of disinformation, such as an initial perceived intent to disinform and content aimed at the general public rather than a specific audience. Other factors, including content presented by licensed professionals such as a counseling resident, an ear-nose-throat surgeon, or a therapist, and country-specific variables from Ireland, Colombia, and the Philippines, as well as topics such as adjustment disorder, addiction, eating disorders, and impulse control disorders, showed a weak negative association with disinformation. In terms of engagement, only the number of favorites was significantly associated with a reduction in disinformation. Five recommendations were made to enhance the quality of educational videos about mental health on social media platforms.

**Conclusions:**

This study is the first to provide specific, data-driven recommendations to mental health providers globally, addressing the current state of disinformation on social media. Further research is needed to assess the implementation of these recommendations by health professionals, their impact on patient health, and the quality of mental health information on social networks.

## Introduction

Mental health is a prevalent topic worldwide on social media platforms [1]. However, fake news and erroneous information on social media platforms are serious issues that considerably impact public trust and health [2,3]. The purposeful spread of incorrect or misleading information with the objective to mislead or influence the public is known as disinformation [4]. This kind of information is frequently disseminated to sway public opinion, hide the truth, or accomplish commercial, military, or political objectives [5]. Disinformation differs from misinformation and is defined as false information accidentally disseminated, without malicious intent [4]. This is different from misinformation that implies a deceptive aim. These falsehoods can take various forms, such as promoting untested remedies or treatments, voicing inaccurate health claims, and propagating conspiracy theories about illnesses, climate change, and vaccines [6]. Due to the viral nature of social media, false information can spread rapidly and widely, potentially influencing people’s attitudes and behaviors related to health [7,8]. This is particularly dangerous during health emergencies, such as pandemics, when accurate information is important for everyone’s safety [8,9].

Health-related disinformation that is widely spread may lead to treatment reluctance, unhealthy lifestyle choices, and mistrust of medical professionals and scientific institutions [10]. Various strategies have been used to attempt to counter these repercussions including fact-checking campaigns, promoting evidence-based content, and forming partnerships between health organizations and social media platforms to highlight credible sources and flag or remove misleading information [11,12]. Despite these efforts, the fight against false health information on social media continues, emphasizing the need for the public to develop critical media literacy skills to navigate and assess the reliability of health-related information on the web [13,14].

Disinformation related to mental health on social media presents specific risks since it feeds stigma, dispels myths, and erodes public awareness of mental health concerns [15]. Myths concerning the etiology of mental diseases and inaccurate information regarding the efficacy of treatments are examples of false information that frequently sensationalizes or minimizes these conditions [16,17]. In addition to maintaining stigma, this may deter people from getting professional assistance or pursuing evidence-based therapies [15]. Such disinformation may have a significant negative impact by exacerbating feelings of loneliness, anxiousness, and depression in populations that are already vulnerable [18]. Social media platforms, mental health experts, and the general public must work together to address disinformation about mental health to generate truthful and accessible information, conduct candid discussions about mental health, and offer assistance to individuals who are impacted by these problems [19]. While ensuring reliable access to science-backed mental health resources and education is important, there are currently no clear recommendations, nor study focusing specifically on this phenomenon that could lead to promoting a better informed and supportive web-based community in the field of mental health.

Many studies are presented in the literature to highlight the impact of disinformation in the social medias on the general public. For example, a systematic review highlighted that social media use is extremely common among youth, and exposure to disinformation can exacerbate mental health issues such as anxiety and depression [20]. Furthermore, the spread of inaccurate information about specific disorders, such as attention-deficit/hyperactivity disorder (ADHD), has been prevalent. A study analyzing the top 100 ADHD-related videos on TikTok found that less than half adhered to clinical guidelines, raising concerns about disinformation leading to self-diagnosis among teenagers [21]. The motivations behind disseminating mental health disinformation on social media are multifaceted. A bibliometric analysis identified various intervention strategies to address disinformation sharing, emphasizing the need for proactive measures to decrease the spread of inaccurate information [22]. Additionally, the misuse of psychological terminology on social media platforms has diluted the meaning of important concepts. Terms such as “gaslighting,” “boundaries,” and “toxic relationships” are often used inaccurately, leading to misunderstandings and minimizing the experiences of those truly affected by such issues [23,24]. Another concerning trend is “sadfishing,” where individuals exaggerate emotional problems on the web to garner sympathy. This behavior can undermine genuine mental health struggles and expose vulnerable users to cyberbullying and exploitation [25]. This explorative study aims to investigate the phenomenon of disinformation related to mental health on social media. The specific objectives are to (1) identify key factors that can predict whether a social media video contains misleading or false information for viewers and listeners, (2) estimate the prevalence of different mental health topics addressed in these videos and determine which topics are most frequently associated with disinformation, and (3) develop recommendations for best practices in the creation and dissemination of psychoeducational videos on mental health aimed at the general public.

Given the limited empirical research on the spread of mental health disinformation on social media, this study adopts an exploratory approach. Nonetheless, we propose several working hypotheses to guide the analysis. First, we hypothesize that disinformation about mental illnesses is prevalent within mental health–related content on social media platforms. Second, we anticipate that certain mental health topics, such as ADHD and depression, are disproportionately represented in videos containing inaccurate or misleading information. Third, we hypothesize that specific content features, including emotional tone, the use of clinical language, and the presence of personal anecdotes, may serve as significant predictors of disinformation. Through this exploratory investigation, the study aims to generate foundational insights that can support the development of evidence-based guidelines and interventions to establish more accurate and supportive mental health communication in digital environments.

## Methods

This observational study analyzed 1000 publicly available TikTok videos across 26 mental health topics in 3 languages using a disinformation coding framework and machine learning. A multilayered perceptron model was applied to identify content features and predictors associated with disinformation.

### Participants and Recruitment

This study did not actively recruit participants or use their data. Instead, it involved the collection and analysis of TikTok videos, which are publicly accessible. TikTok was chosen as the primary source of information due to its widespread use as a social media platform and the public nature of its content [26]. The research team identified a total of 1000 videos based on the following inclusion criteria: (1) the video must address a mental health topic (directly reference psychological or psychiatric conditions, mental health, mental well-being, or treatment or diagnostic processes); (2) it must contain psychoeducational material or be intended to be psychoeducational; (3) the language must be French, English, or Spanish (as these are the languages the coders are fluent with); (4) the video must not be part of a multipart series (this was applied to ensure the independence and self-containment of each unit of analysis); and (5) the video must be accessible without requiring a TikTok account.

It is important to note that videos were not collected via keyword searches alone, but we did identify candidate videos by monitoring popular hashtags and search trends associated with mental health (eg, #ADHD, #mentalhealth, #therapy, #anxiety, #depression, and #mentalhealthawareness). The For You page was then explored to curate a broader sample. Importantly, no filters were applied based on the source of the video. That is, videos created by verified organizations (eg, World Health Organization and Centers for Disease Control and Prevention) and individual creators (eg, influencers, lay users, and self-identified professionals) were all considered, provided they met the inclusion criteria. Accounts clearly operating as bots or spam (eg, repetitive posting with identical content, commercial-only links, or suspicious usernames) were flagged and excluded during a manual screening process. Video length was not used as an inclusion or exclusion criterion; however, excessively short videos (<5 seconds) that did not contain any identifiable mental health–related content were removed.

Given that web users represent approximately 65.1% of the global population, we used this proportion as a reference point to estimate an appropriate sample size for our exploratory analysis. Assuming a confidence level of 95% and a 5% margin of error, parameters commonly used in social science research, a minimum sample size of 385 videos was determined to be sufficient to provide statistically meaningful insights into the broader population of mental health content encountered on social media. This calculation was conducted using G*Power 3.1, based on a proportion test (*z* test) for goodness of fit to ensure adequate representation and to inform descriptive and inferential components of the study [27]. The platform’s “For You” tab was used, which curates content using TikTok’s in-house recommendation algorithm based on popular topics, user engagement, and relevance, to gather the sample of 1000 TikTok videos. This strategy was chosen to represent the kind of content that users are most likely to come across naturally. We sought to obtain a genuine and ecologically valid representation of commonly seen mental health–related information by using the algorithmically curated For You feed instead of just hashtags or keyword searches. To reduce personalization bias and get a wide variety of videos from different topics and producers, the research team used freshly made, neutral accounts with no past engagement history for several sampling sessions.

### Ethical Considerations

The ethics committee of the Centre de recherche de l’Institut universitaire en santé mentale de Montréal was consulted to determine the need for ethics approval for this study’s objectives and methodology. According to the Canadian Tri-Council Policy Statement on Ethical Conduct for Research Involving Humans, no ethics board approval was required, as the TikTok videos analyzed belong to the public domain. This study was registered and can be found in Multimedia Appendix 1, as well as the dataset used.

### Data Collection

A dataset was collaboratively constructed by all the authors, identifying key elements from 3 different categories for each analyzed video: video characteristics, quality of the information extracted, and clinical elements related to mental health. The data collected in this study are shown in Table 1. Weighted numbers for likes, comments, shares, and favorites were obtained by dividing their total amount by the number of days elapsed since the upload date.

**Table 1.** Categories and variables collected for each identified TikTok video.

Categories	Elements
Video characteristics	Video upload date Country Professional title of the presenter (self-reported title) Language (language) Lengths (in minutes) Weighted number of likes Weighted number of comments Weighted number of shares Weighted number of favorites Target audience (general or specific audience) Hashtags used
Quality of the information	Content (opinion-based, fact-based, and mixed) Intent (disinformation, misinformation, clickbait, satire, and other) Authenticity (rumor, propaganda, hoax, conspiracy, framing, reference-based, and other)
Clinical mental health data	Topic (main topic) Reference used

The elements related to the quality of the information are inspired by the work of Aïmeur and colleagues [28], who introduced formal definitions of content, intent, and authenticity in the context of fake news. In this study, content can be classified as opinion-based, fact-based, or a mix of both (mixed). The intent refers to the perceived intent of the video as analyzed by the researcher. It can be categorized as disinformation (providing false information or information not supported by the scientific community), misinformation (information that is partially correct according to the scientific community), clickbait (with the main intent to sell a product or promote a service), satire (using humor or sketches to convey information), or other (when the intent appears to inform, educate, or provide a personal experience, and the information does not fall into disinformation or misinformation). Authenticity refers to the source of information conveyed in the video. The sources can be classified as a rumor, propaganda, a hoax, a conspiracy, framing (using part of the information out of context or using humor or sketches as the source of information), reference-based (mentioning or citing explicit sources of information), or other.

The members of the research team (n=8) received training in order to uniformize the data collection. Each video was counter-validated by another member of the research team so that all videos have been assessed at least twice. Prior to full coding, the team conducted a pilot test on a random subsample of 50 videos to calibrate interpretation and resolve discrepancies. Interrater reliability was calculated using Cohen κ for categorical decisions (eg, inclusion and topic classification), and overall agreement reached 87%, indicating a strong level of consistency. Disagreements were resolved through group discussion until consensus was reached.

### Data Analysis

Observational descriptive statistics, such as the identified prevalence of each variable, were reported. Correlations between the collected elements and disinformation were established using Spearman ρ, as the data did not follow a normal distribution. To assess the variables most linked to disinformation, a machine learning approach using a multilayered perceptron classifier from the open-source scikit-learn library (Python 3.9) was used to account for the number of variables and multicollinearity [29].

The neural network architecture included 2 hidden layers with 64 and 32 neurons, respectively, using the rectified linear unit activation function. Categorical variables were converted into accessible variables using a one-hot encoding approach. The independent variables were defined as all columns of the dataset except the disinformation column, which was designated as the dependent variable. To train the classifier, the dataset was split into training and testing sets using a standard 80‐20 split to ensure that the model could generalize to unseen data. The model was then cross-validated using the 10-fold algorithm from scikit-learn to ensure consistency in the reported performance [29]. Model accuracy was assessed using the *F*_1_-score, and odds ratios, *P* values, and CIs were reported for each independent variable. The *F*_1_-score, commonly used in text classification, provides a balanced measure of theme classification accuracy by combining both precision and recall into a single metric [30]. A *P* value of less than .05 was considered significant.

Finally, recommendations were established based on the elements that were found to be significant and strongly correlated with either the presence or the absence of disinformation. The recommendations were also driven by relevant literature. Considering the observational nature of this study, the STROBE (Strengthening the Reporting of Observational Studies in Epidemiology) statement was used to report the findings.

## Results

A total of 1000 TikTok videos were analyzed. The vast majority were in English (n=830), of unspecified geographic origin (n=618), and presented by individuals whose titles were not provided (n=471). Disinformation was most frequent in English language videos in absolute terms; however, when adjusted for sample size, French language videos showed a proportionally higher presence of disinformation. Topic prevalence also varied by language: English videos more commonly addressed ADHD, anxiety, and depression, while French videos focused on psychotherapy and personality disorders, and Spanish videos often covered emotional regulation and general mental well-being. Although English videos received the highest engagement overall, French language videos flagged for disinformation showed relatively high engagement within their language group, suggesting that misinformation in less represented languages may have a concentrated impact. These findings underscore the importance of considering linguistic and cultural dimensions when addressing mental health disinformation on social media platforms. Notably, among the professionals, most of the videos were created by psychiatrists and psychologists. The videos were primarily aimed at the general public rather than a specific audience. The video-related elements are summarized in Table 2.

**Table 2.** Video-related variables of identified TikTok videos regarding sociodemographic characteristics.

Variable	Values, n (%)	
Language		
English	830 (83.00)	
French	108 (10.8)	
Spanish	62 (6.2)	
Country		
Not specified	618 (61.8)	
United States	236 (23.6)	
United Kingdom	41 (4.1)	
Canada	41 (4.1)	
France	27 (2.7)	
Australia	18 (1.8)	
Mexico	3 (0.3)	
Sweden	3 (0.3)	
Australia	2 (0.2)	
India	2 (0.2)	
Philippines	2 (0.2)	
Ecuador	2 (0.2)	
Peru	1 (0.1)	
Belgium	1 (0.1)	
Colombia	1 (0.1)	
Ireland	1 (0.1)	
Spain	1 (0.1)	
Presented by (professional title)		
Not specified	471 (47.1)	
Psychiatrist	131 (13.1)	
Psychologist	73 (7.3)	
Therapist	56 (5.6)	
Coach	25 (2.5)	
Nurse	24 (2.4)	
Medical resident	19 (1.9)	
Doctor	15 (1.5)	
Influencer	14 (1.4)	
Family doctor	7 (0.7)	
Pediatrician	7 (0.7)	
Other	158 (15.8)	
Targeted audience		
General public	896 (89.6)	
Specific audience	104 (10.4)	

When assessing the topics of the videos and their engagement metrics, it was observed that most of the identified videos were about personality disorders, autism, depression, and ADHD. The longest videos focused on personality disorders, while the shortest video addressed dissociative identity disorder. Videos on addiction and autism received the highest engagement, as indicated by the average weighted number of likes, comments, favorites, and shares. It is to be noted that the mental health topic included any mental health–related subject matter that was not specific to a diagnosis or treatment. The topics and engagement metrics are summarized in Table 3.

**Table 3.** Topics and engagement metrics for each mental health topic identified in the TikTok videos.

Mental health topic	Values, n (%)		Average video length (minutes)	Average weighted number of likes	Average weighted number of comments	Average weighted number of favorites	Average weighted number of shares
Personality disorders	207 (20.7)		1.83	1123	31	206	86
Autism	90 (9.00)		1.73	5406	106	979	1081
Depression	90 (9.00)		0.92	3691	89	526	560
ADHD^a^	90 (9.00)		1.37	1897	17	251	74
Psychotic disorders	82 (8.2)		1.77	428	11	53	25
Anxiety	81 (8.1)		1.15	880	11	130	112
Mental health	76 (7.6)		1.04	1774	25	284	179
Treatment	69 (6.9)		1.30	200	17	40	24
Bipolar disorders	46 (4.6)		1.30	218	3	28	10
Trauma	34 (3.4)		1.23	3048	41	623	94
OCD^b^	20 (2.00)		0.90	218	3	24	18
Psychotherapy	15 (1.5)		1.66	903	12	163	89
Psychiatry	14 (1.4)		1.53	450	5	53	13
Eating disorders	14 (1.4)		1.11	994	6	48	15
Suicide	13 (1.3)		1.39	432	7	56	11
Tourette syndrome	11 (1.1)		1.03	35	3	2	2
Impulse control disorders	9 (0.9)		1.66	17	1	4	3
Somatization	8 (0.8)		1.08	63	3	9	6
Neurocognitive disorders	8 (0.8)		1.31	5	0	1	0
Neurodevelopmental	5 (0.5)		1.13	124	24	20	22
Addiction	5 (0.5)		1.78	17,196	337	1869	1148
Adjustment disorder	4 (0.4)		1.36	85	83	3	21
Sleep disorders	4 (0.4)		0.67	1213	21	216	98
Catatonia	2 (0.2)		1.43	28	0	3	0
Dissociative identity disorder	2 (0.2)		0.33	1514	20	122	33
Paraphilia	1 (0.1)		1.33	4	0	0	0

a ADHD: attention-deficit/hyperactivity disorder.

b OCD: obsessive-compulsive disorder.

Across the various mental health topics, most videos were opinion-based, with 6.30% (63/1000) containing disinformation and 15.70% (157/1000) containing misinformation. Reference-based videos represented 20.70% (207/1000) of the analyzed content. Neurodevelopmental disorders, suicide, and mental health were the topics for which the videos were predominantly opinion-based, followed closely by dissociative identity disorder, autism, and depression. Videos on neurodevelopment, mental health, personality disorders, suicide, psychotic disorders, and treatment conveyed the highest amounts of disinformation. In videos discussing neurodevelopmental disorders, particularly ADHD and autism, disinformation often took the form of oversimplified self-diagnosis criteria (eg, “If you forget your keys, you definitely have ADHD”) or misleading statements about cures through dietary supplements. In the mental health category, disinformation included generalized claims such as “mental illness is just a mindset” or “you don’t need therapy, just positive thinking,” which risk minimizing serious conditions. Videos addressing personality disorders frequently portrayed traits of borderline or narcissistic personality disorder using inaccurate or stigmatizing descriptions, often lacking any clinical basis. In content related to suicide, misinformation included videos that romanticized suicidal ideation or presented recovery without professional intervention as universally effective. For psychotic disorders, we found cases where hallucinations were portrayed as spiritual awakenings or entirely controllable through willpower. Finally, videos on treatment sometimes promoted unverified therapies or discouraged the use of psychiatric medication with claims such as “antidepressants only make things worse” or “therapy is a scam.” The content, intent, and authenticity for each topic are shown in Multimedia Appendix 1.

Correlations of all variables regarding disinformation are shown in Multimedia Appendix 2. Overall, a weak positive correlation exists between opinion-based content and disinformation, as well as sources listed as propaganda, rumor, hoax, or conspiracy. Certain professional titles, such as brain health or brainspotting practitioner, were also weakly correlated with disinformation. On the other hand, videos with the intent to inform or educate, with various sources of information, that are fact-based and presented by psychiatrists were correlated negatively with disinformation.

When modeling the dataset to predict disinformation as per the variables pertaining to quality of the information, only the intent to misinform (odds ratio [OR] 1.07, 95% CI 1.02‐1.11) and content directed to general public (OR 1.04, 95% CI 1.01‐1.07) were significantly associated with disinformation. Other variables, such as presented by a licensed resident in counseling (OR 0.95, 95% CI 0.93‐0.98), an ear-nose-throat surgeon (OR 0.96, 95% CI 0.93‐0.98), and a therapist (OR 0.96, 95% CI 0.93‐0.98); country-specific variables such as Ireland (OR 0.98, 95% CI 0.95‐1.00), Colombia (OR 0.97, 95% CI 0.95‐0.99), and Philippines (OR 0.97, 95% CI 0.94‐0.99); and specific topics such as adjustment disorder (OR 0.97, 95% CI 0.94‐0.99), addiction (OR 0.97, 95% CI 0.94‐1.00), eating disorders (OR 0.97, 95% CI 0.95‐0.99), and impulse control disorders (OR 0.97, 95% CI 0.95‐1.00) were weakly inversely associated with disinformation. As for engagement, only the number of favorites was significantly associated negatively with disinformation (OR 0.97, 95% CI 0.95‐1.00). The *F*_1_-score of the model was 89.19%, with an adjusted *R*^2^ score of 0.79. Coefficients for each variable used in the model are shown in Multimedia Appendix 3.

## Discussion

### Principal Results

The aim of this study was to explore the phenomenon of disinformation about mental health on social media. A total of 1000 TikTok videos, coming from more than 16 countries, in 3 languages (English, French, and Spanish) and encompassing a total of 26 topics were thoroughly analyzed. Disinformation was mostly found in videos discussing neurodevelopment, mental health, personality disorders, suicide, psychotic disorders, and treatment. A machine learning model allowed identification of weak predictors of disinformation, such as an initial perceived intent to misinform or information provided to the general public rather than a specific audience. Other factors, such as content presented by a licensed counseling resident, an ear-nose-throat surgeon, or a therapist, as well as country-specific variables like those from Ireland, Colombia, and the Philippines, and specific topics, such as adjustment disorder, addiction, eating disorders, and impulse control disorders, showed a weak negative association with disinformation. Regarding engagement, only the number of favorites was significantly associated with less disinformation.

Misinformation and disinformation are commonly observed on social media platforms [31,32]. This was also observed in this study, considering that 6.30% and 15.70% of the videos were perceived as having an intent to disinform or misinform the viewers. This phenomenon is important to consider because it can have several consequences on viewers, especially those with mental health–related vulnerabilities, even more so in periods of stress [33,34]. Namely, a recent review examining the impact of fake news in the health sector reported that fake news in the context of COVID-19 can lead to psychological disorders and induce panic, fear, depression, and fatigue [2]. Since engaging with social media content (such as likes, comments, and followers) may contribute to poor mental health, information vehiculated by professionals on these platforms should be of high quality [35,36]. Professionals in the mental health field are beginning to use web-based technologies in their work, but opinions on the morality and practicality of doing so in clinical settings still vary considering the heterogeneity of content available to the public on social media [37].

While professional guidelines on the use of social media exist for general health care professionals, no specific recommendations exist regarding the creation of content on social media for mental health professionals [38]. Such guidelines are needed considering many of the observed correlations with disinformation. For instance, opinion-based videos, which were the most prevalent type of videos identified in this study, are to be dealt with carefully. Considering their association with disinformation, mental health professionals should refrain from conveying solely opinion-based content as they may be misinterpreted by the viewers. The lack of reference-based content observed in this study also aligns with observations made in the literature regarding general health social media content. The following recommendations are based on the content that has been analyzed in the context of this study. Mental health professionals who wish to limit disinformation and its potential adverse effects may consider them when creating social media content.

### Theoretical and Practical Contributions

This study offers both theoretical insight and practical guidance in an area that has received limited empirical attention. On a theoretical level, it contributes to the understanding of how disinformation circulates in the context of mental health, a topic that presents particular challenges due to stigma, emotional sensitivity, and the complexity of psychiatric diagnoses. By examining a large, multilingual sample of social media content and applying a machine learning approach to identify patterns associated with disinformation, the study provides empirical support for previously assumed associations such as the influence of audience targeting and professional credibility. This adds to the current literature by grounding these assumptions in observational data rather than anecdotal or speculative claims. From a practical standpoint, a set of evidence-informed recommendations for mental health professionals who use social media to engage with the public is found in the following subsection. These guidelines are based directly on observed patterns in the data and aim to help clinicians communicate more clearly, avoid common pitfalls, and reduce the spread of misleading or inaccurate information. By doing so, the study not only highlights gaps in current practices but also offers a starting point for improving the quality of mental health content on the web.

### Recommendations to Mental Health Professionals Creating Social Media Content

#### Target a Specific Audience

It is recommended to state the intended audience at the beginning of the video to ensure that its content can be interpreted correctly by the viewers [39,40]. Videos may be customized, as well as the material conveyed, to meet the unique requirements, interests and preferences of the specific audience. This increases the audience’s relevance and engagement with your communications, which raises the possibility of a favorable response while limiting interpretation biases.

#### Use and Cite Relevant Sources

As observed in the correlation analysis, fact-based resources and intent to inform were less associated with disinformation. Videos will be more credible if reliable sources are properly credited [41]. Doing so not only demonstrates that the presented material is supported by scientific studies and validated by professionals but also allows to build trust with the audience and permits fact-checking by interested viewers. It is also a way to provide the audience with a mean to understand potential biases in the information presented.

#### State Your Credentials

Given the heterogeneity in the demographics of social media content creators and that many professional titles had a correlation with disinformation in this study, it is important to provide users with your credentials [42]. This can include the country from which the information is presented, your professional title, and a summary of your experience. This is important to take into consideration, since several guidelines for various topics in mental health may differ depending on the country or the jurisdiction in which they were developed. This provides helpful information to the viewers to better understand the context in which the information is provided.

#### State Your Intent and Use Fact-Based Content

Explicitly stating the intent of the video and relying on fact-based content rather than opinion are less associated with disinformation [43,44]. To avoid confusion of the viewership or misinterpretation, opinion-based content should be stated as such so that the viewers can critically appreciate the information vehiculated.

#### Avoid Oversimplifying Complex Topics

Topics such as autism, personality disorders, and psychotic disorders were correlated with disinformation, and it can be hypothesized that this is because they are complex topics. Generalization of these subjects should be avoided to limit misinterpretation of the information transmitted to the viewers [45,46]. Breaking down complex topics into smaller units could be a way to better convey the information.

As an example, to support the implementation of these recommendations, mental health professionals can leverage specific tools and strategies already available on social media platforms. Creators could use captioning tools or video intros to clearly state their target audience and credentials within the first few seconds of a video. Platforms such as TikTok also allow creators to include link-in-bio tools or descriptions where references and source material can be cited. Institutions or professional associations can contribute by providing media literacy training or tool kits tailored for clinicians, outlining how to communicate responsibly on social media. Collaborative efforts with science communicators or digital health experts may further help translate evidence-based practices into accessible video formats. Additionally, integrating these best practices into professional guidelines or continuing education programs may normalize and incentivize responsible content creation among mental health providers.

### Limitations

This study has a few limitations. As with many machine learning models, challenges arise when interpreting the meaning of the correlations identified for each variable. For example, some variables that might be expected to correlate strongly with disinformation, such as fact-based content, did not show statistically significant associations in the model. This can be attributed to the small number of fact-based videos identified, potentially introducing biases during model training. Therefore, the interpretation of the model should be cautiously based only on the significant variables and also the fact that the data were solely extracted from TikTok. Model overfitting is commonly observed in multilayered classifiers, and it is hypothesized that the significance of certain variables could vary as the dataset grows. Important steps, such as data cleaning and standardization, were undertaken to mitigate overfitting as much as possible for this dataset. The intent and authenticity of the videos were assessed based on the evaluators’ judgment, as contacting individual content creators was beyond the scope of this study. However, 2 independent evaluations of each video were done in order to limit potential erroneous interpretations of its content.

### Conclusions

This study aimed to explore the characteristics and prevalence of disinformation in TikTok videos related to mental health and to identify factors associated with this phenomenon. By analyzing 1000 publicly available videos across multiple countries and languages, the study offers a comprehensive and data-driven view of how disinformation manifests in social media content related to psychiatric topics. Important patterns were identified, including topics and content types most vulnerable to disinformation, and video characteristics (such as lack of references or unspecified professional titles) that may contribute to the spread of misleading information.

Beyond describing these patterns, the study also contributes theoretically by applying an observational analytic framework and machine learning model to a largely unexamined area: mental health disinformation in user-generated content. This methodological approach allowed for the identification of a few weak, yet, statistically significant predictors of disinformation, advancing empirical understanding of how various factors interact in web-based health communication. These findings respond directly to gaps in the existing literature, where disinformation in mental health contexts has often been discussed anecdotally or in isolated case studies, without systematic or scalable analysis.

From a practical standpoint, this study is among the first to translate such findings into specific, actionable recommendations for mental health professionals. These guidelines are grounded in empirical observation and are intended to help clinicians communicate more clearly, reduce misinterpretation, and contribute to a more informed web-based discourse. Given the growing role of social media in contributing to the public understanding of mental health, these contributions are both timely and necessary.

Although this study focused on TikTok, the insights generated could be applicable to other social media platforms that rely on short-form content and algorithm-driven visibility. As such, the findings support the development of broader communication guidelines and professional training initiatives, with the aim of strengthening the quality and reliability of mental health information on the web. Offering both methodological innovation and applied recommendations, this study brings a new and valuable perspective to the ongoing challenge of health-related disinformation in the digital age.

## Supplementary material

10.2196/64225 Multimedia Appendix 1 Content, intent, and authenticity count percentages per topic.

10.2196/64225 Multimedia Appendix 2 Summary of correlations of all variables in relation with disinformation.

10.2196/64225 Multimedia Appendix 3 Machine learning classifier’s variables and their coefficients for each variable to predict disinformation.

10.2196/64225 Checklist 1 STROBE (Strengthening the Reporting of Observational Studies in Epidemiology) checklist.

## Abbreviations

ADHD: attention-deficit/hyperactivity disorder
STROBE: Strengthening the Reporting of Observational Studies in Epidemiology
