# Supplementary material for: Navigating the Maze of Social Media Disinformation on Psychiatric Illness and Charting Paths to Reliable Information for Mental Health Professionals: Observational Study of TikTok Videos
Source: J Med Internet Res. 2025 Jun 18;27:e64225. doi: 10.2196/64225 (PMC12192922; doi:10.2196/64225)
Supplement: Multimedia Appendix 1 [file jmir-v27-e64225-s001.docx]

Supplementary information on this study is available at <doi:10.17605/OSF.IO/FS7BX>.
The full dataset is available at: <https://osf.io/a9672>. Links to all the videos are available upon reasonable request.

**Multimedia Appendix 1.** Content, intent, and authenticity count percentages per topic.

| **Topic** | **Number** | **Content (Opinion-based: %, Fact-based: %, Mixed: %)** | **Intent (Disinformation : % , Misinformation: %, Clickbait: %, Satire: %, Other: %)** | **Authenticity (Rumor: %, Propaganda: %, Hoax: %, Conspiracy: %, Framing: %, Reference-based: %, Other: %)** |
| --- | --- | --- | --- | --- |
| Personality disorders | 207 | 33.82,27.54,38.65 | 8.21,24.15,3.38,3.38,60.87 | \| 9.18,0.48,0.00,0.48,22.71,18.84,48.31 \| \| --- \| |
| Autism | 90 | 42.22,23.33,34.44 | 4.44,22.22,2.22,7.78,63.33 | \| 10.00,1.11,7.78,1.11,13.33,10.00,56.67 \| \| --- \| |
| Depression | 90 | 41.11,28.89,30.00 | 4.44,22.22,7.78,5.56,60.00 | \| 8.89,3.33,1.11,0.00,15.56,21.11,50.00 \| \| --- \| |
| ADHD | 90 | 38.89,30.00,31.11 | 3.33,15.56,3.33,10.00,67.78 | \| 13.33,0.00,6.67,1.11,14.44,7.78,56.67 \| \| --- \| |
| Psychotic disorders | 82 | 28.05,51.22,20.73 | 7.32,8.54,2.44,3.66,78.05 | \| 4.88,2.44,1.22,3.66,3.66,41.46,42.68 \| \| --- \| |
| Anxiety | 81 | 49.38,18.52,32.10 | 9.88,16.05,9.88,4.94,59.26 | \| 13.58,2.47,2.47,3.70,11.11,14.81,51.85 \| \| --- \| |
| Mental health | 76 | 61.84,10.53,27.63 | 15.79,9.21,7.89,6.58,60.53 | \| 14.47,3.95,2.63,1.32,15.79,7.89,53.95 \| \| --- \| |
| Treatment | 69 | 17.39,52.17,30.43 | 7.25,4.35,10.14,4.35,73.91 | \| 1.45,2.90,4.35,0.00,8.70,37.68,44.93 \| \| --- \| |
| Bipolar disorder | 46 | 21.74,36.96,41.30 | 2.17,19.57,2.17,4.35,71.74 | \| 2.17,0.00,0.00,0.00,6.52,26.09,65.22 \| \| --- \| |
| Trauma | 34 | 38.24,20.59,41.18 | 2.94,17.65,8.82,2.94,67.65 | \| 8.82,5.88,0.00,5.88,17.65,20.59,41.18 \| \| --- \| |
| OCD | 20 | 30.00,30.00,40.00 | 0.00,10.00,5.00,25.00,60.00 | \| 0.00,0.00,0.00,0.00,25.00,15.00,60.00 \| \| --- \| |
| Psychotherapy | 15 | 20.00,33.33,46.67 | 0.00,0.00,0.00,33.33,66.67 | \| 0.00,0.00,0.00,0.00,13.33,20.00,66.67 \| \| --- \| |
| Psychiatry | 14 | 35.71,42.86,21.43 | 0.00,0.00,7.14,21.43,71.43 | \| 0.00,7.14,0.00,0.00,21.43,7.14,64.29 \| \| --- \| |
| Eating disorders | 14 | 14.29,50.00,35.71 | 0.00,0.00,7.14,0.00,92.86 | \| 0.00,0.00,0.00,0.00,7.14,28.57,64.29 \| \| --- \| |
| Suicide | 13 | 69.23,7.69,23.08 | 7.69,0.00,7.69,0.00,84.62 | \| 0.00,7.69,0.00,0.00,38.46,7.69,46.15 \| \| --- \| |
| Tourette syndrome | 11 | 18.18,63.64,18.18 | 0.00,18.18,0.00,9.09,72.73 | \| 0.00,0.00,0.00,0.00,9.09,18.18,72.73 \| \| --- \| |
| Impulse control disorder | 9 | 0.00,55.56,44.44 | 0.00,22.22,11.11,0.00,66.67 | \| 0.00,0.00,11.11,0.00,0.00,44.44,44.44 \| \| --- \| |
| Somatization | 8 | 0.00,62.50,37.50 | 0.00,12.50,0.00,0.00,87.50 | \| 0.00,0.00,0.00,0.00,12.50,62.50,25.00 \| \| --- \| |
| Neurocognitive disorder | 8 | 0.00,87.50,12.50 | 0.00,0.00,0.00,0.00,100.00 | \| 0.00,0.00,0.00,0.00,0.00,75.00,25.00 \| \| --- \| |
| Neurodevelopmental | 5 | 80.00,0.00,20.00 | 20.00,0.00,0.00,40.00,40.00 | \| 0.00,20.00,0.00,0.00,0.00,0.00,80.00 \| \| --- \| |
| Addiction | 5 | 20.00,40.00,40.00 | 0.00,0.00,0.00,0.00,100.00 | \| 0.00,0.00,0.00,0.00,0.00,0.00,100.00 \| \| --- \| |
| Adjustment disorder | 4 | 0.00,50.00,50.00 | 0.00,0.00,0.00,25.00,75.00 | \| 0.00,0.00,0.00,0.00,0.00,75.00,25.00 \| \| --- \| |
| Sleep disorder | 4 | 0.00,75.00,25.00 | 0.00,25.00,0.00,0.00,75.00 | \| 0.00,0.00,0.00,0.00,25.00,50.00,25.00 \| \| --- \| |
| Catatonia | 2 | 0.00,50.00,50.00 | 0.00,0.00,0.00,0.00,100.00 | \| 0.00,0.00,0.00,0.00,0.00,50.00,50.00 \| \| --- \| |
| Dissociative identity disorder | 2 | 50.00,0.00,50.00 | 0.00,0.00,50.00,0.00,50.00 | \| 0.00,0.00,0.00,0.00,100.00,0.00,0.00 \| \| --- \| |
| Paraphilia | 1 | 0.00,100.00,0.00 | 0.00,0.00,0.00,0.00,100.00 | \| 0.00,0.00,0.00,0.00,0.00,100.00,0.00 \| \| --- \| |
| **Total** | 1000 | 35.80,31.40,32.80 | 6.30,15.70,5.20,6.30,66.50 | 7.90,1.90,2.30,1.20,14.60,20.70,51.40 |

Acronyms: ADHD: attention deficit with hyperactivity disorder; OCD: obsessive-compulsive disorder
