# Supplementary material for: Navigating the Maze of Social Media Disinformation on Psychiatric Illness and Charting Paths to Reliable Information for Mental Health Professionals: Observational Study of TikTok Videos
Source: J Med Internet Res. 2025 Jun 18;27:e64225. doi: 10.2196/64225 (PMC12192922; doi:10.2196/64225)
Supplement: Multimedia Appendix 2 [file jmir-v27-e64225-s002.docx]

**Multimedia Appendix 2.** Summary of correlations of all variables in relation with disinformation.

| **Variable** | **Correlation with Disinformation** |
| --- | --- |
| Intent Disinformation | 1.0 |
| Authenticity Propaganda | 0.24 |
| Content Opinion | 0.23 |
| Presented by Ph.D in brain health | 0.17 |
| Presented by Brainspotting Practitioner | 0.17 |
| Authenticity Rumor | 0.15 |
| Authenticity Hoax | 0.15 |
| Authenticity Conspiracy | 0.12 |
| Target audience Therapists | 0.12 |
| Presented by Psychology diploma. Master's degree in social work | 0.12 |
| Presented by Hormone. thyroid and gut imbalance specialist | 0.12 |
| Presented by psychotherapist | 0.12 |
| Target audience People with autism spectrum disorder | 0.11 |
| Presented by Holistic psychiatrist | 0.11 |
| Topic Mental health | 0.11 |
| Presented by Not specified | 0.1 |
| Presented by Doctor | 0.07 |
| Authenticity Framing | 0.07 |
| Presented by Researcher | 0.06 |
| Language English | 0.05 |
| Target audience People with ADHD | 0.05 |
| Topic Anxiety | 0.04 |
| Topic Personality disorders | 0.04 |
| Topic Neurodevelopmental | 0.04 |
| Presented by Influencer | 0.04 |
| Target audience General public | 0.03 |
| Country United Kingdom | 0.03 |
| Country Not specified | 0.03 |
| Topic Psychotic disorders | 0.01 |
| Topic Treatment | 0.01 |
| Country France | 0.01 |
| Topic Suicide | 0.01 |
| Country Australia | -0.0 |
| Length (minutes) | -0.01 |
| Presented by Somatic Experiencing Practitioner | -0.01 |
| Presented by Certified Clinical Trauma Specialist and a Narcissistic Abuse Recovery Professional. | -0.01 |
| Presented by Somatic hypnotherapist | -0.01 |
| Target audience People with eating disorders | -0.01 |
| Target audience People with OCD | -0.01 |
| Presented by Nutritionnist | -0.01 |
| Presented by Ph.D in developmental psychology/neuroscience | -0.01 |
| Target audience People with a neurodivergence | -0.01 |
| Target audience Children | -0.01 |
| Presented by Licensed resident in counseling | -0.01 |
| Presented by Licensed psychometrician | -0.01 |
| Presented by Mental health worker and advocate | -0.01 |
| Presented by Heart surgeon | -0.01 |
| Presented by Intervention agent | -0.01 |
| Presented by Artist | -0.01 |
| Presented by Cosmetic surgeon | -0.01 |
| Presented by Licensed clinical social worker | -0.01 |
| Presented by psychologist | -0.01 |
| Country Peru | -0.01 |
| Presented by ICU medical doctor | -0.01 |
| Topic Paraphilia | -0.01 |
| Presented by Holistic nutritionist | -0.01 |
| Target audience People who use cannabis | -0.01 |
| Presented by Ph.D. in clinical counseling | -0.01 |
| Presented by Holistic paediatrician | -0.01 |
| Target audience People without ADHD | -0.01 |
| Country Brussels | -0.01 |
| Presented by Massage therapist | -0.01 |
| Target audience People who experienced trauma | -0.01 |
| Presented by Psychology student | -0.01 |
| Presented by Actor | -0.01 |
| Presented by Sophrologist | -0.01 |
| Presented by Certificate of Clinical Competence in Speech Language Pathology | -0.01 |
| Target audience People suffering from internet gaming disorder | -0.01 |
| Presented by Holistic psychologist. hynotherapist | -0.01 |
| Presented by Plastic surgeon | -0.01 |
| Presented by Anesthesiologist | -0.01 |
| Presented by School principal | -0.01 |
| Country Colombia | -0.01 |
| Presented by Founder and CEO of cord blood banking company | -0.01 |
| Presented by Neuroscience Ph.D. | -0.01 |
| Presented by Streamer. Youtuber | -0.01 |
| Presented by Medical Doctor | -0.01 |
| Country Spain | -0.01 |
| Presented by Hairstylist | -0.01 |
| Country Ireland | -0.01 |
| Country United States of America | -0.01 |
| Language French | -0.01 |
| Topic Catatonia | -0.01 |
| Presented by Neuroscientist | -0.01 |
| Presented by Journalist | -0.01 |
| Presented by Biologist | -0.01 |
| Presented by Speaker | -0.01 |
| Presented by Pharmacist | -0.01 |
| Country Ecuador | -0.01 |
| Country Philippines | -0.01 |
| Presented by Integrative somatic therapy practice | -0.01 |
| Topic Dissociative identity disorder | -0.01 |
| Presented by Neuropsychologist | -0.01 |
| Presented by ENT surgeon | -0.01 |
| Presented by Student | -0.01 |
| Presented by Obstetrician-gynecologist | -0.01 |
| Presented by M.S. Psychology. Certified autism specialist | -0.01 |
| Presented by Ophthalmologist | -0.01 |
| Presented by neuropsychologist | -0.01 |
| Country Australia | -0.01 |
| Presented by Clinical Homeopath | -0.01 |
| Country India | -0.01 |
| Presented by Guru | -0.01 |
| Presented by Advisor | -0.01 |
| Presented by Teacher | -0.01 |
| Presented by Psychotherapist | -0.01 |
| Country Sweden | -0.01 |
| Presented by Ph.D Candidate. M.A. Psychology | -0.01 |
| Presented by Comedian | -0.01 |
| Country Mexico | -0.01 |
| Presented by Medical Student | -0.01 |
| Presented by Coach | -0.02 |
| Weighted Comments | -0.02 |
| Presented by Counselor | -0.02 |
| Topic Adjustment disorder | -0.02 |
| Presented by Medical student | -0.02 |
| Presented by Psychopracticioner | -0.02 |
| Presented by Actor | -0.02 |
| Presented by therapist | -0.02 |
| Topic Sleep disorder | -0.02 |
| Presented by Psychology student | -0.02 |
| Target audience People with depression | -0.02 |
| Weighted Share | -0.02 |
| Presented by M.A. Psychology. Ph.D candidate | -0.02 |
| Topic Addiction | -0.02 |
| Presented by Social worker | -0.02 |
| Presented by Neurologist | -0.02 |
| Presented by Neuroscientist | -0.02 |
| Presented by Podcaster | -0.02 |
| Weighted Favorites | -0.02 |
| Presented by Family doctor | -0.02 |
| Presented by Medical doctor | -0.02 |
| Target audience People with borderline personality disorders | -0.02 |
| Presented by Paediatrician | -0.02 |
| Topic Somatization | -0.02 |
| Topic Neurocognitive disorder | -0.02 |
| Topic Depression | -0.02 |
| Topic Autism | -0.02 |
| Topic Impulse control disorder | -0.02 |
| Presented by Psychologist | -0.03 |
| Topic Trauma | -0.03 |
| Target audience People with anxiety | -0.03 |
| Topic Tourette syndrome | -0.03 |
| Presented by Therapist | -0.03 |
| Weighted likes | -0.03 |
| Topic Eating disorders | -0.03 |
| Presented by psychiatrist | -0.03 |
| Topic Psychiatry | -0.03 |
| Topic Psychotherapy | -0.03 |
| Presented by Not specified | -0.03 |
| Presented by Medical resident | -0.04 |
| Topic OCD | -0.04 |
| Topic Bipolar disorder | -0.04 |
| Topic ADHD | -0.04 |
| Presented by Nurse | -0.04 |
| Country Canada | -0.05 |
| Intent Clickbait | -0.06 |
| Language Spanish | -0.07 |
| Content Mixed | -0.07 |
| Intent Satire | -0.07 |
| Target audience Parents | -0.07 |
| Presented by Psychiatrist | -0.08 |
| Intent Misinformation | -0.11 |
| Authenticity Reference-based | -0.11 |
| Content Fact-based | -0.17 |
| Authenticity Other | -0.18 |
| Intent Other | -0.37 |

Acronyms: ADHD: attention deficit with hyperactivity disorder; OCD: obsessive-compulsive disorder, ENT: Ear, nose, throat, ICU: Intensive care unit.
