# Supplementary material for: Navigating the Maze of Social Media Disinformation on Psychiatric Illness and Charting Paths to Reliable Information for Mental Health Professionals: Observational Study of TikTok Videos
Source: J Med Internet Res. 2025 Jun 18;27:e64225. doi: 10.2196/64225 (PMC12192922; doi:10.2196/64225)
Supplement: Multimedia Appendix 3 [file jmir-v27-e64225-s003.docx]

**Multimedia Appendix 3.** Machine learning classifier’s variables and their coefficients for each variable to predict disinformation.

| **Variables** | **Coefficient** | **OR^a^** | **2.5% CI** | **97.5% CI** | ***P* value** |
| --- | --- | --- | --- | --- | --- |
| Intent Misinformation | 0.07 | 1.07 | 1.02 | 1.11 | .003 |
| Intent Other | 0.06 | 1.06 | 1.01 | 1.12 | .073 |
| Target audience General public | 0.04 | 1.04 | 1.01 | 1.07 | .005 |
| Presented by Not specified | 0.03 | 1.03 | 1.00 | 1.06 | .038 |
| Country Not specified | 0.03 | 1.03 | 1.00 | 1.05 | .046 |
| Authenticity Other | 0.02 | 1.02 | 0.99 | 1.05 | .133 |
| Topic Personality disorders | 0.02 | 1.02 | 1.00 | 1.04 | .065 |
| Presented by Therapist | 0.02 | 1.02 | 0.99 | 1.04 | .217 |
| Topic Psychotic disorders | 0.02 | 1.02 | 0.99 | 1.04 | .134 |
| Topic Depression | 0.01 | 1.01 | 0.99 | 1.04 | .218 |
| Authenticity Framing | 0.01 | 1.01 | 0.99 | 1.04 | .352 |
| Authenticity Rumor | 0.01 | 1.01 | 0.99 | 1.04 | .371 |
| Content Fact-based | 0.01 | 1.01 | 0.98 | 1.04 | .474 |
| Content Mixed | 0.01 | 1.01 | 0.98 | 1.04 | .564 |
| Presented by Guru | 0.01 | 1.01 | 0.98 | 1.03 | .693 |
| Presented by Comedian | 0.00 | 1.00 | 0.98 | 1.03 | .735 |
| Presented by Mental health worker and advocate | 0.00 | 1.00 | 0.98 | 1.03 | .830 |
| Presented by Psychologist | 0.00 | 1.00 | 0.98 | 1.02 | .922 |
| Topic ADHD^b^ | 0.00 | 1.00 | 0.98 | 1.02 | .940 |
| Presented by Nurse | 0.00 | 1.00 | 0.98 | 1.03 | .959 |
| Presented by Neuroscientist | 0.00 | 1.00 | 0.98 | 1.02 | .973 |
| Presented by Medical Doctor | 0.00 | 1.00 | 1.00 | 1.00 | .195 |
| Country Brussels | 0.00 | 1.00 | 1.00 | 1.00 | .294 |
| Presented by Sophrologist | 0.00 | 1.00 | 1.00 | 1.00 | .725 |
| Presented by Ph.D. in clinical counseling | 0.00 | 1.00 | 1.00 | 1.00 | .738 |
| Presented by Founder and CEO of cord blood banking company | 0.00 | 1.00 | 1.00 | 1.00 | .506 |
| Presented by actor | 0.00 | 1.00 | 1.00 | 1.00 | .268 |
| Topic Autism | 0.00 | 1.00 | 0.97 | 1.03 | .962 |
| Language English | 0.00 | 1.00 | 0.97 | 1.03 | .960 |
| Presented by Intervention agent | 0.00 | 1.00 | 1.00 | 1.00 | .086 |
| Length (minutes) | 0.00 | 1.00 | 0.98 | 1.02 | .941 |
| Topic Mental health | 0.00 | 1.00 | 0.97 | 1.02 | .913 |
| Language French | 0.00 | 1.00 | 0.97 | 1.02 | .867 |
| Topic Anxiety | 0.00 | 1.00 | 0.97 | 1.02 | .856 |
| Country United Kingdom | 0.00 | 1.00 | 0.98 | 1.02 | .818 |
| Topic Neurodevelopmental | 0.00 | 1.00 | 0.98 | 1.02 | .816 |
| Intent Clickbait | 0.00 | 1.00 | 0.96 | 1.03 | .839 |
| Topic Bipolar disorder | 0.00 | 1.00 | 0.97 | 1.02 | .775 |
| Authenticity Reference-based | 0.00 | 1.00 | 0.97 | 1.02 | .728 |
| Presented by Massage therapist | 0.00 | 1.00 | 0.97 | 1.02 | .750 |
| Presented by Coach | −0.01 | 0.99 | 0.97 | 1.02 | .692 |
| Country Canada | −0.01 | 0.99 | 0.97 | 1.02 | .687 |
| Presented by Not specified | −0.01 | 0.99 | 0.97 | 1.02 | .677 |
| Intent Satire | −0.01 | 0.99 | 0.96 | 1.03 | .725 |
| Presented by Ophthalmologist | −0.01 | 0.99 | 0.97 | 1.02 | .622 |
| Language Spanish | −0.01 | 0.99 | 0.97 | 1.02 | .608 |
| Presented by Pharmacist | −0.01 | 0.99 | 0.96 | 1.02 | .490 |
| Presented by MA Psychology. PhD candidate | −0.01 | 0.99 | 0.96 | 1.02 | .491 |
| Weighted Share | −0.01 | 0.99 | 0.97 | 1.02 | .465 |
| Target audience People with anxiety | −0.01 | 0.99 | 0.96 | 1.02 | .508 |
| Presented by Integrative somatic therapy practice | −0.01 | 0.99 | 0.96 | 1.02 | .491 |
| Target audience People with depression | −0.01 | 0.99 | 0.97 | 1.01 | .389 |
| Topic Psychiatry | −0.01 | 0.99 | 0.96 | 1.02 | .437 |
| Target audience People with borderline personality disorders | −0.01 | 0.99 | 0.96 | 1.02 | .427 |
| Target audience People with eating disorders | −0.01 | 0.99 | 0.96 | 1.02 | .436 |
| Country Spain | −0.01 | 0.99 | 0.96 | 1.01 | .411 |
| Content Opinion | −0.01 | 0.99 | 0.96 | 1.01 | .410 |
| Authenticity Hoax | −0.01 | 0.99 | 0.97 | 1.01 | .380 |
| Country Australia | −0.01 | 0.99 | 0.96 | 1.01 | .407 |
| Presented by Hairstylist | −0.01 | 0.99 | 0.96 | 1.02 | .429 |
| Topic Suicide | −0.01 | 0.99 | 0.96 | 1.02 | .434 |
| Target audience People with autism spectrum disorder | −0.01 | 0.99 | 0.96 | 1.01 | .393 |
| Presented by Family doctor | −0.01 | 0.99 | 0.96 | 1.01 | .360 |
| Authenticity Conspiracy | −0.01 | 0.99 | 0.96 | 1.01 | .379 |
| Country United States of America | −0.01 | 0.99 | 0.96 | 1.01 | .323 |
| Target audience People without ADHD | −0.01 | 0.99 | 0.96 | 1.02 | .385 |
| Presented by Doctor | −0.01 | 0.99 | 0.96 | 1.01 | .330 |
| Presented by Certified Clinical Trauma Specialist and a Narcissistic Abuse Recovery Professional. | −0.01 | 0.99 | 0.96 | 1.02 | .418 |
| Country France | −0.01 | 0.99 | 0.97 | 1.01 | .292 |
| Presented by Teacher | −0.01 | 0.99 | 0.96 | 1.01 | .326 |
| Presented by Psychopractitioner | −0.01 | 0.99 | 0.96 | 1.01 | .312 |
| Topic Dissociative identity disorder | −0.01 | 0.99 | 0.96 | 1.01 | .297 |
| Presented by Psychotherapist | −0.01 | 0.99 | 0.96 | 1.01 | .322 |
| Target audience People who use cannabis | −0.01 | 0.99 | 0.96 | 1.01 | .278 |
| Presented by School principal | −0.01 | 0.99 | 0.96 | 1.01 | .263 |
| Presented by Licensed psychometrician | −0.01 | 0.99 | 0.96 | 1.01 | .289 |
| Presented by Counselor | −0.01 | 0.99 | 0.96 | 1.01 | .277 |
| Presented by Medical resident | −0.02 | 0.98 | 0.96 | 1.02 | .321 |
| Presented by psychology student | −0.02 | 0.98 | 0.96 | 1.01 | .216 |
| Topic Trauma | −0.02 | 0.98 | 0.96 | 1.01 | .280 |
| Presented by Anesthesiologist | −0.02 | 0.98 | 0.96 | 1.01 | .202 |
| Presented by Psychology student | −0.02 | 0.98 | 0.96 | 1.01 | .194 |
| Topic OCD^c^ | −0.02 | 0.98 | 0.96 | 1.01 | .188 |
| Weighted likes | −0.02 | 0.98 | 0.96 | 1.01 | .197 |
| Topic Paraphilia | −0.02 | 0.98 | 0.96 | 1.01 | .170 |
| Presented by Artist | −0.02 | 0.98 | 0.96 | 1.01 | .164 |
| Presented by Neurologist | −0.02 | 0.98 | 0.96 | 1.01 | .146 |
| Topic Treatment | −0.02 | 0.98 | 0.96 | 1.00 | .108 |
| Country Peru | −0.02 | 0.98 | 0.96 | 1.01 | .130 |
| Target audience. People with OCD | −0.02 | 0.98 | 0.96 | 1.01 | .159 |
| Topic Somatization | −0.02 | 0.98 | 0.96 | 1.01 | .141 |
| Country India | −0.02 | 0.98 | 0.96 | 1.01 | .172 |
| Presented by Licensed clinical social worker | −0.02 | 0.98 | 0.96 | 1.01 | .126 |
| Presented by Hormone. Thyroid and gut imbalance specialist | −0.02 | 0.98 | 0.96 | 1.01 | .151 |
| Target audience. People with a neurodivergence | −0.02 | 0.98 | 0.95 | 1.01 | .212 |
| Presented by Psychiatrist | −0.02 | 0.98 | 0.95 | 1.01 | .184 |
| Presented by Influencer | −0.02 | 0.98 | 0.96 | 1.00 | .108 |
| Topic Sleep disorder | −0.02 | 0.98 | 0.95 | 1.01 | .158 |
| Presented by Social worker | −0.02 | 0.98 | 0.96 | 1.00 | .094 |
| Target audience. Children | −0.02 | 0.98 | 0.95 | 1.01 | .165 |
| Country Mexico | −0.02 | 0.98 | 0.95 | 1.01 | .115 |
| Presented by Somatic hypnotherapist | −0.02 | 0.98 | 0.95 | 1.01 | .148 |
| Presented by PhD in developmental psychology/neuroscience | −0.02 | 0.98 | 0.95 | 1.00 | .095 |
| Presented by Advisor | −0.02 | 0.98 | 0.95 | 1.00 | .081 |
| Presented by PhD in brain health | −0.02 | 0.98 | 0.95 | 1.00 | .107 |
| Presented by Podcaster | −0.02 | 0.98 | 0.95 | 1.01 | .124 |
| Presented by psychiatrist | −0.02 | 0.98 | 0.95 | 1.00 | .089 |
| Presented by Certificate of Clinical Competence in Speech Language Pathology | −0.02 | 0.98 | 0.95 | 1.00 | .102 |
| Country Sweden | −0.02 | 0.98 | 0.95 | 1.00 | .072 |
| Presented by Streamer. Youtuber | −0.02 | 0.98 | 0.95 | 1.00 | .084 |
| Weighted Comments | −0.02 | 0.98 | 0.95 | 1.00 | .087 |
| Target audience. Therapists | −0.02 | 0.98 | 0.95 | 1.00 | .082 |
| Target audience. Parents | −0.02 | 0.98 | 0.95 | 1.00 | .101 |
| Topic Psychotherapy | −0.02 | 0.98 | 0.95 | 1.00 | .048 |
| Presented by psychologist | −0.02 | 0.98 | 0.95 | 1.00 | .072 |
| Presented by Holistic psychiatrist | −0.02 | 0.98 | 0.95 | 1.00 | .058 |
| Presented by Student | −0.02 | 0.98 | 0.95 | 1.00 | .076 |
| Country Ecuador | −0.02 | 0.98 | 0.95 | 1.00 | .065 |
| Presented by Holistic pediatrician | −0.02 | 0.98 | 0.95 | 1.00 | .092 |
| Presented by Biologist | −0.02 | 0.98 | 0.95 | 1.00 | .047 |
| Presented by Pediatrician | −0.02 | 0.98 | 0.95 | 1.00 | .079 |
| Presented by Clinical Homeopath | −0.02 | 0.98 | 0.95 | 1.00 | .086 |
| Presented by Holistic psychologist. Hynotherapist | −0.02 | 0.98 | 0.95 | 1.00 | .079 |
| Topic Catatonia | −0.03 | 0.98 | 0.95 | 1.00 | .069 |
| Country Ireland | −0.03 | 0.98 | 0.95 | 1.00 | .043 |
| Topic Neurocognitive disorder | −0.03 | 0.97 | 0.95 | 1.00 | .060 |
| Presented by Speaker | −0.03 | 0.97 | 0.95 | 1.00 | .048 |
| Presented by Neuroscience PhD | −0.03 | 0.97 | 0.95 | 1.00 | .043 |
| Country Australia | −0.03 | 0.97 | 0.95 | 1.00 | .072 |
| Target audience. People who experienced trauma | −0.03 | 0.97 | 0.95 | 1.00 | .050 |
| Presented by Psychology diploma. Master’s degree in social work | −0.03 | 0.97 | 0.95 | 1.00 | .028 |
| Weighted Favorites | −0.03 | 0.97 | 0.95 | 1.00 | .037 |
| Topic Impulse control disorder | −0.03 | 0.97 | 0.95 | 1.00 | .048 |
| Target audience. People with ADHD | −0.03 | 0.97 | 0.95 | 1.00 | .048 |
| Presented by neuropsychologist | −0.03 | 0.97 | 0.95 | 1.00 | .036 |
| Presented by Obstetrician—gynecologist | −0.03 | 0.97 | 0.95 | 1.00 | .030 |
| Presented by Researcher | −0.03 | 0.97 | 0.95 | 1.00 | .021 |
| Presented by Medical Student | −0.03 | 0.97 | 0.95 | 1.00 | .039 |
| Presented by Plastic surgeon | −0.03 | 0.97 | 0.95 | 1.00 | .026 |
| Authenticity Propaganda | −0.03 | 0.97 | 0.95 | 0.99 | .017 |
| Target audience. People suffering from internet gaming disorder | −0.03 | 0.97 | 0.95 | 0.99 | .015 |
| Presented by neuroscientist | −0.03 | 0.97 | 0.95 | 0.99 | .017 |
| Topic Eating disorders | −0.03 | 0.97 | 0.95 | 0.99 | .015 |
| Presented by Heart surgeon | −0.03 | 0.97 | 0.95 | 1.00 | .019 |
| Presented by psychotherapist | −0.03 | 0.97 | 0.94 | 1.00 | .039 |
| Presented by Journalist | −0.03 | 0.97 | 0.95 | 0.99 | .018 |
| Presented by Actor | −0.03 | 0.97 | 0.94 | 1.00 | .042 |
| Topic Tourette syndrome | −0.03 | 0.97 | 0.94 | 1.00 | .025 |
| Topic Addiction | −0.03 | 0.97 | 0.94 | 1.00 | .029 |
| Presented by Holistic nutritionist | −0.03 | 0.97 | 0.94 | 0.99 | .018 |
| Country Colombia | −0.03 | 0.97 | 0.95 | 0.99 | .008 |
| Presented by Nutritionist | −0.03 | 0.97 | 0.95 | 0.99 | .009 |
| Presented by Medical doctor | −0.03 | 0.97 | 0.94 | 0.99 | .016 |
| Country Philippines | −0.03 | 0.97 | 0.94 | 0.99 | .012 |
| Presented by Cosmetic surgeon | −0.03 | 0.97 | 0.94 | 0.99 | .009 |
| Presented by Medical student | −0.03 | 0.97 | 0.94 | 0.99 | .008 |
| Presented by Brainspotting Practitioner | −0.03 | 0.97 | 0.94 | 0.99 | .112 |
| Presented by therapist | −0.04 | 0.97 | 0.94 | 0.99 | .012 |
| Topic Adjustment disorder | −0.04 | 0.97 | 0.94 | 0.99 | .005 |
| Presented by MS Psychology. Certified autism specialist | −0.04 | 0.96 | 0.94 | 0.99 | .005 |
| Presented by Neuropsychologist | −0.04 | 0.96 | 0.94 | 0.99 | .010 |
| Presented by Somatic Experiencing Practitioner | −0.04 | 0.96 | 0.94 | 0.99 | .003 |
| Presented by ICU^d^ medical doctor | −0.04 | 0.96 | 0.94 | 0.99 | .004 |
| Presented by therapist | −0.04 | 0.96 | 0.93 | 0.98 | .002 |
| Presented by ENT^e^ surgeon | −0.04 | 0.96 | 0.93 | 0.98 | .001 |
| Presented by Licensed resident in counseling | −0.05 | 0.95 | 0.93 | 0.98 | .001 |

^a^OR: odds ratio.

^b^ADHD: attention-deficit/hyperactivity disorder.

^c^OCD: obsessive-compulsive disorder.

^d^ICU: intensive care unit.

^e^ENT: ear-nose-throat.
